# Comparison of Six‐ and Three‐Dimensional Error Corrections for the Planning Target Volume in Patients With Nasopharyngeal Cancer

**DOI:** 10.1002/jmrs.70126

**Published:** 2026-09-18

**Authors:** Huan‐Chun Chen, Ming‐Chung Chou

**Affiliations:** ^1^ Department of Radiation Oncology Kaohsiung Municipal Ta‐Tung Hospital Kaohsiung Taiwan; ^2^ Department of Medical Imaging and Radiological Sciences Kaohsiung Medical University Kaohsiung Taiwan; ^3^ Biomedical Artificial Intelligence Academy Kaohsiung Medical University Kaohsiung Taiwan; ^4^ Center for Big Data Research Kaohsiung Medical University Kaohsiung Taiwan; ^5^ Department of Medical Research Kaohsiung Medical University Hospital Kaohsiung Taiwan

**Keywords:** CBCT, nasopharyngeal cancer, radiation therapy, six‐dimensional correction

## Abstract

**Introduction:**

During radiation therapy, patients may experience positioning errors that can affect the irradiated volume and result in unnecessary radiation doses to normal tissues. Correcting for setup errors is essential for patients undergoing radiation therapy; however, it remains unclear whether six‐dimensional (6D) correction is superior to conventional three‐dimensional (3D) correction in nasopharyngeal cancer. Therefore, the purpose of this study was to compare the planning target volume (PTV) coverage between 3D and 6D cone‐beam computed tomography (CBCT) corrections for patients with nasopharyngeal carcinoma.

**Methods:**

We retrospectively enrolled 30 patients with nasopharyngeal cancer who underwent image‐guided radiation therapy. The 3D and 6D corrections were performed using CBCT images registered to CT simulation images based on skeletal structures at 1, 3, and 5 weeks, respectively. The volume percentages of the PTV achieving the prescribed dose of 70, 63, and 56 Gy were calculated after both 3D and 6D corrections and were statistically compared using a repeated measures analysis of variance test.

**Results:**

The results showed that the percentage coverage of the target volume was 94.1% ± 4.3% and 95.4% ± 3.4% for 70 Gy, 94.4% ± 2.6% and 95.3% ± 2.4% for 63 Gy, and 92.1% ± 7.7% and 95.6% ± 5.4% for 56 Gy after 3D and 6D corrections, respectively. A significant difference was noted in the volume coverage for 56 Gy between the two correction methods. Additionally, the average maximum dose percentages after 3D and 6D corrections were 5.9% ± 8.0% and 3.3% ± 6.2% for the spinal cord, and 1.8% ± 8.5% and 2.7% ± 6.0% for the brain stem, respectively; however, no significant difference was observed between the two correction methods.

**Conclusion:**

We conclude that 6D correction for CBCT images achieved better dose coverage of the target tumour volume than 3D correction in patients with nasopharyngeal carcinoma.

## Graphical abstract

6D correction for CBCT achieves better dose coverage of target tumour volume than 3D correction in patients with nasopharyngeal carcinoma. 6D correction significantly improved the dose coverage of PTV‐low at 5 weeks after treatment as compared to 3D correction.

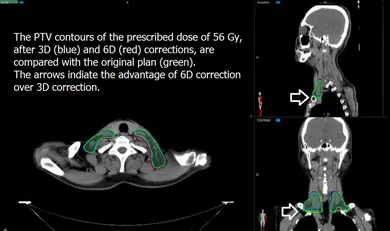

## 1 Introduction

Nasopharyngeal carcinoma (NPC) is more prevalent in regions such as Southeast China, Hong Kong, Taiwan, Singapore, and North Africa than other parts of the world [1, 2, 3], and the main treatment methods include radiation therapy, chemotherapy, and concurrent or sequential chemoradiotherapy. According to the GLOBOCAN 2022 report, there were over 120,000 new NPC cases and 73,000 deaths annually worldwide, with the incidence in male subjects being more than twice that of female subjects (86,257 vs. 34,159, respectively) [4]. Radiation therapy is the standard treatment for patients with early‐stage NPC, while chemoradiotherapy is typically used for those with advanced NPC and lymph node metastases [5, 6].

Radiation therapy utilizes high‐energy X‐rays for irradiation, necessitating a high level of accuracy and reproducibility. Patients may experience positioning errors that can affect the irradiation of the tumour site and result in unnecessary exposure to normal tissues. These errors for each treatment fraction can be corrected using image guidance [7]. While common linear accelerators can identify six‐dimensional (6D) error data, including translations and rotations along the *X*, *Y*, and *Z* axes, most systems are limited to performing only three‐dimensional (3D) translational corrections due to constraints in the treatment table hardware [8].

Some previous studies applied 6D correction of cone‐beam computed tomography (CBCT) to address intra‐fractional motion in stereotactic body radiation therapy, demonstrating that this approach enhances the precision and safety of radiosurgery treatments [9, 10, 11]. One previous study further utilized 6D CBCT correction to reduce setup errors in radiation therapy for intracranial head and neck tumours [12]. Although their results indicated that 6D correction did not provide a distinct advantage over conventional 3D correction, it was noted that 6D correction was more beneficial for patients with larger tumour sizes [12]. In NPC, the anatomical location of the nasopharynx is adjacent to the sphenoid bone, which means that setup errors may adversely affect dose coverage of the target volume and increase the maximum dose to neighbouring critical organs. Therefore, minimizing setup errors in radiation therapy for NPC patients using 6D correction is essential.

In recent years, advancements in radiation therapy equipment have resulted in linear accelerators being equipped with 6D correction treatment tables designed to reduce positioning errors, enhance treatment accuracy, improve tumour control probability, and decrease the risk of complications to normal tissues. However, to the best of our knowledge, no prior study has evaluated the impact of 3D and 6D corrections on dose coverage of the target volume and dose to critical organs in radiation therapy for NPC patients. Therefore, the purpose of this study was to compare the differences in planning target volume (PTV) coverage of nasopharyngeal tumours and the maximum dose percentage to critical organs between 3D and 6D corrections using CBCT images.

## 2 Materials and Methods

This retrospective study was approved by the local institutional review board of Kaohsiung Medical University Hospital (protocol number: KMUHIRB‐E(II)‐20220040), and the informed consent was waived due to the retrospective nature of the study. This study retrospectively collected data from September 2017 to March 2021 involving nasopharyngeal cancer patients who received image‐guided radiation therapy. All patients were immobilized with head rest and thermoplastic mask during the radiation therapy, and a re‐setup was performed if translation > 3 mm or rotation angle > 3 degrees. Patients who required re‐simulation (including re‐scanning CT and re‐planning) due to significant weight loss or changes in anatomical structure were excluded from the study. A total of 30 patients were successfully enrolled in this study. Among them, 15 were treated with 3D correction, while the remaining patients received 6D correction for CBCT images. To understand the impact of correction method on dose distribution, the CBCT images were corrected using 3D and 6D registration techniques, respectively.

Among these patients, there were 22 males and 8 females, with ages ranging from 34 to 86 years and a mean age of 56 years. Staging was performed according to the standards of the American Joint Committee on Cancer, resulting in 1 patient at Stage 0, 2 at Stage I, 5 at Stage II, 14 at Stage III, and 8 at Stage IV. Of the patients, 27 received concurrent chemotherapy, while 3 did not undergo chemotherapy. After treatment, 7 patients experienced recurrence. The demographic characteristics of the enrolled patients are presented in Table 1.

**TABLE 1:** The demographics of enrolled patients.

Patients	Number or value
Male	22
Female	8
Age (years)	56 (34–86)
Clinical stage	
0	1
I	2
II	5
III	14
IV	8
Chemotherapy	
Yes	27
No	3
Recurrence	
Yes	7
No	23

For treatment planning, the range of computed tomography simulation (CT‐sim) extended from the orbits to the clavicle, with a slice thickness of 3 mm. During the treatment course, the CBCT images were obtained before irradiation with the same coverage as the CT‐sim, and the CBCT images of treatment fractions at 1, 3 and 5 weeks were used for comparisons. Subsequently, the 3D and 6D corrections were performed, respectively, to register CBCT images to the CT‐sim images using the XVI system version 5.0.2 (Elekta, Stockholm, Sweden) [13]. Specifically, the 3D correction addressed translational errors in the left–right direction (*X*‐axis), superior–inferior direction (*Y*‐axis), and anterior–posterior direction (*Z*‐axis), while the 6D correction accounted for both translational and rotational errors (Pitch, Roll, Yaw) across the *X*, *Y*, and *Z* axes. After rigid image registration was completed, the 3D‐ and 6D‐corrected CBCT images were created using the image fusion system (Velocity, Varian, USA).

Afterwards, the 3D‐ and 6D‐corrected CBCT images were transferred to the treatment planning system (Pinnacle, Philips, The Netherlands) and applied to the original treatment plan's conditions and parameters. The intensity‐modulated radiation therapy planning was performed to evaluate the coverage of the target tumour and organs at risk (OAR) following the 3D and 6D corrections at 1, 3 and 5 weeks, respectively. The volume percentage of the target tumour receiving the prescribed dose (% > max) was assessed to evaluate coverage for doses of 70 Gy (PTV‐high), 63 Gy (PTV‐mid), and 56 Gy (PTV‐low) at 1, 3, and 5 weeks, respectively. Additionally, the maximum dose of the OAR was evaluated based on the percentage of the maximum dose (Dmax) for statistical analysis before and after 3D and 6D corrections at 1, 3 and 5 weeks, respectively.

A repeated measures analysis of variance (ANOVA) was conducted on the dose coverage rates of the target tumour and OAR to assess the sequential differences between 3D and 6D corrections at 1, 3, and 5 weeks. Fisher's Least Significant Difference (LSD) post hoc test was performed for further analysis, with a corrected *p*‐value of < 0.05 considered statistically significant. All statistical analyses were conducted using SPSS Version 22.0 (IBM Corporation, Armonk, NY, USA).

## 3 Results

**FIGURE 1:**
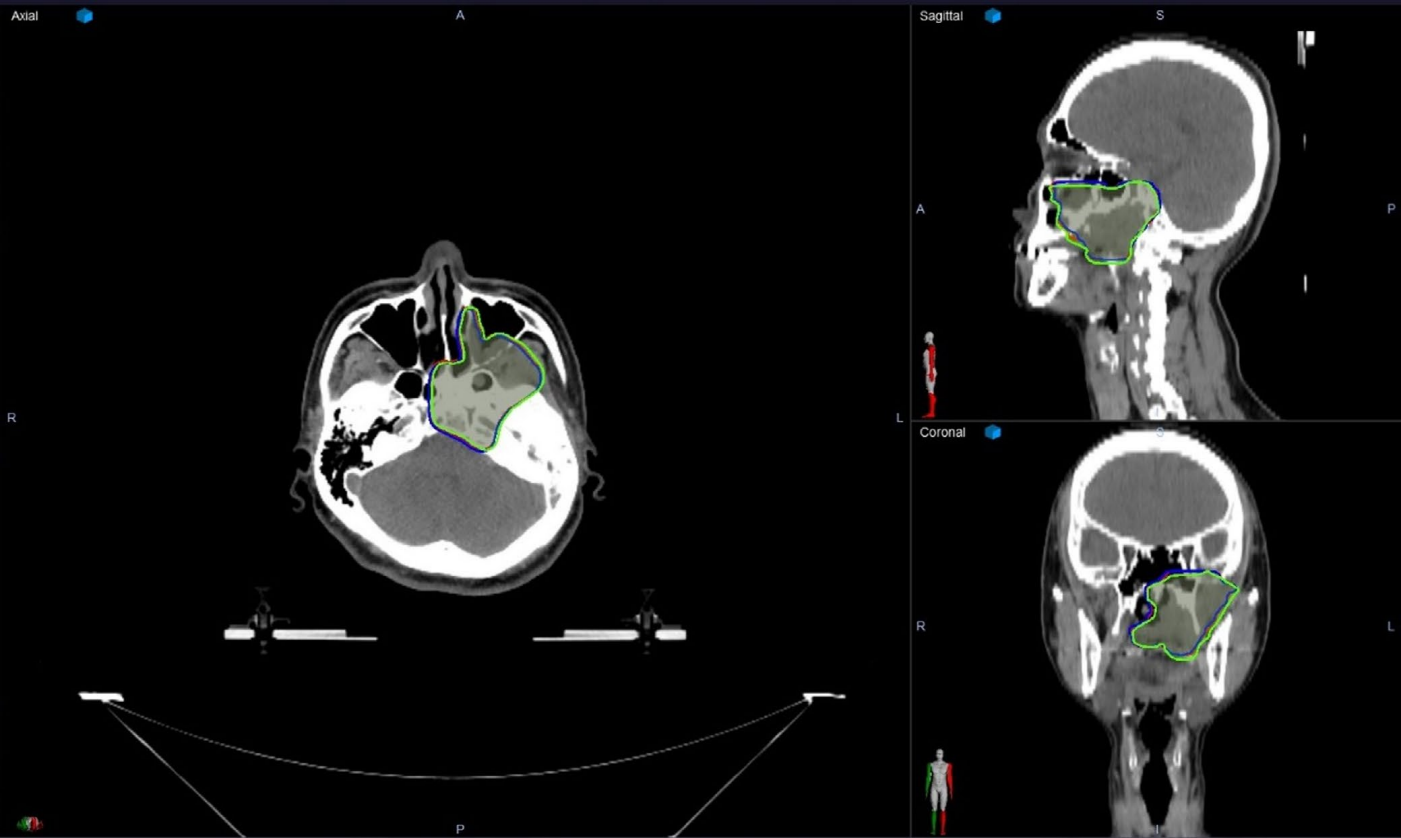
The PTV contours of the prescribed dose of 70 Gy, after 3D (blue) and 6D (red) corrections, are compared with the original plan (green) for a 54‐year‐old male patient with nasopharyngeal cancer at 5 weeks.

The average volume percentages achieving the 70 Gy prescribed dose before and after 3D and 6D corrections compared to CT‐sim treatment planning are shown in Table 2. The ANOVA analysis demonstrated significant differences in the percentage of target volumes receiving prescribed doses of 70 Gy before and after 3D and 6D corrections (*p* = 0.003). The LSD post hoc multiple comparisons further revealed significant differences before and after 3D and 6D corrections, but no significant difference was noted between the 3D and 6D corrections. Figure 1 shows the example of PTV‐high contours after 3D and 6D corrections in a patient with nasopharyngeal cancer. It was noted that the PTV‐high contour after 6D correction had better conformity with the original plan than that after 3D correction at 5 weeks.

**TABLE 2:** The volume percentages achieving the 70, 63 and 56 Gy before and after 3D and 6D corrections at 1, 3 and 5 weeks in NPC patients.

	1 week	3 weeks	5 weeks
70 Gy			
Before	91.8% ± 6.9%*	90.9% ± 6.3%*	91.7% ± 5.6%*
3D	93.9% ± 4.6%*	94.2% ± 4.6%*	94.2% ± 3.7%*
6D	96.0% ± 2.7%*	95.3% ± 3.4%*	94.8% ± 4.1%*
63 Gy			
Before	93.2% ± 3.5%*	92.0% ± 3.5%*	92.8% ± 2.8%*
3D	94.5% ± 2.7%*	94.6% ± 2.5%*	94.1% ± 2.6%*
6D	95.7% ± 2.2%*	94.8% ± 2.5%*	95.5% ± 2.5%*
56 Gy			
Before	92.4% ± 6.1%*	90.4% ± 6.9%*	91.7% ± 5.4%*
3D	92.8% ± 7.6%	91.9% ± 7.1%	91.7% ± 8.5%#
6D	95.8% ± 5.0%*	95.3% ± 4.9%*	95.6% ± 6.4%*#

*Note:* Asterisk symbols (*) indicate significant difference before and after corrections. Hash symbols (#) indicate significant difference between 3D and 6D corrections.

**FIGURE 2:**
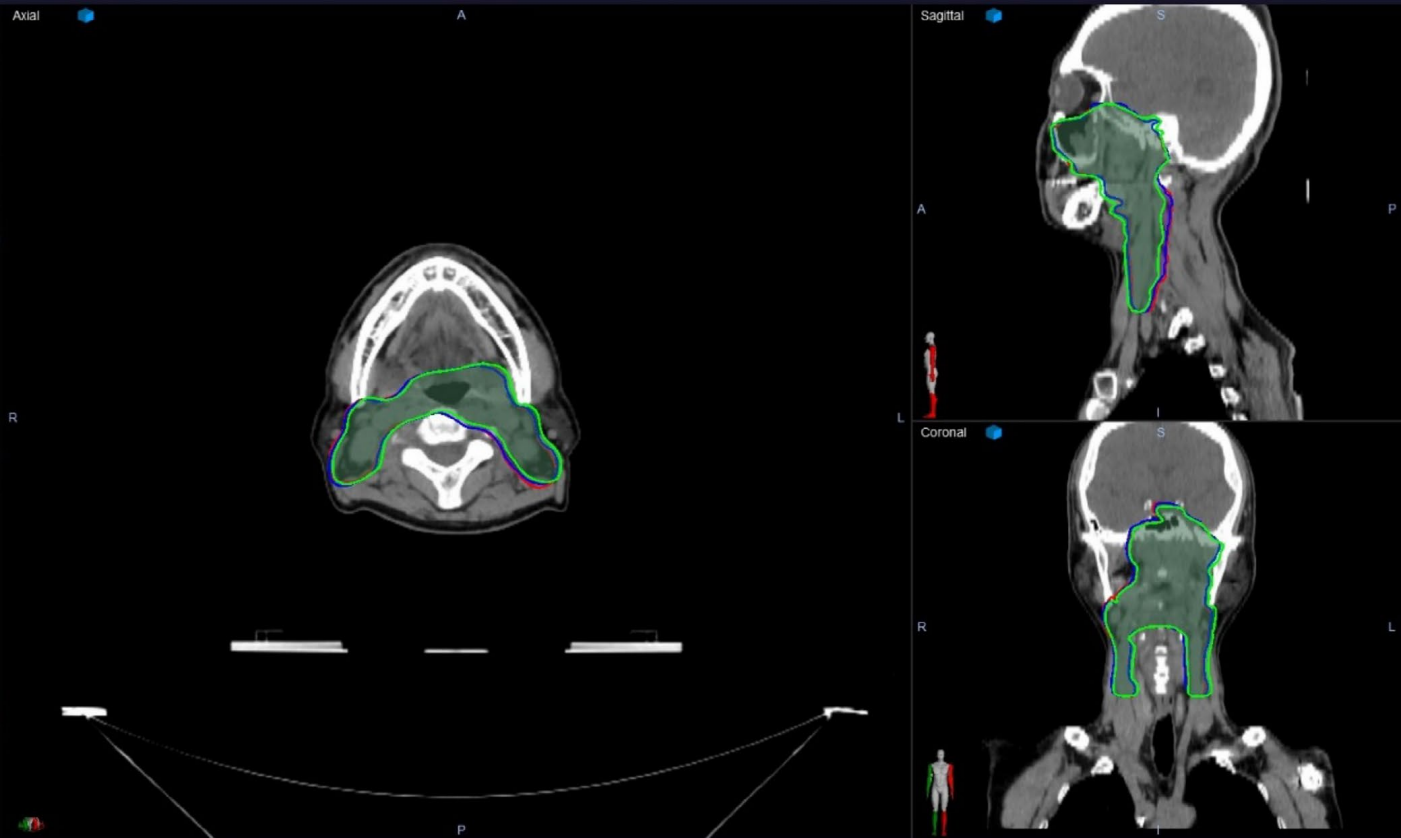
The PTV contours of the prescribed dose of 63 Gy, after 3D (blue) and 6D (red) corrections, are compared with the original plan (green) for a 54‐year‐old male patient with nasopharyngeal cancer at 5 weeks.

The average volume percentages achieving the 63 Gy prescribed dose before and after 3D and 6D corrections compared to CT‐sim treatment planning are shown in Table 2. The ANOVA analysis showed significant differences in the percentage of target volumes receiving prescribed doses of 63 Gy before and after 3D and 6D corrections (*p* = 0.000). The LSD post hoc multiple comparisons further revealed significant differences before and after 3D and 6D corrections, but no significant difference was noted between the 3D and 6D corrections. Figure 2 shows the example of PTV‐mid contours after 3D and 6D corrections in a patient with nasopharyngeal cancer. The comparison demonstrated that the PTV‐mid contour after 6D correction exhibited higher conformity with the original plan than that after 3D correction at 5 weeks.

**FIGURE 3:**
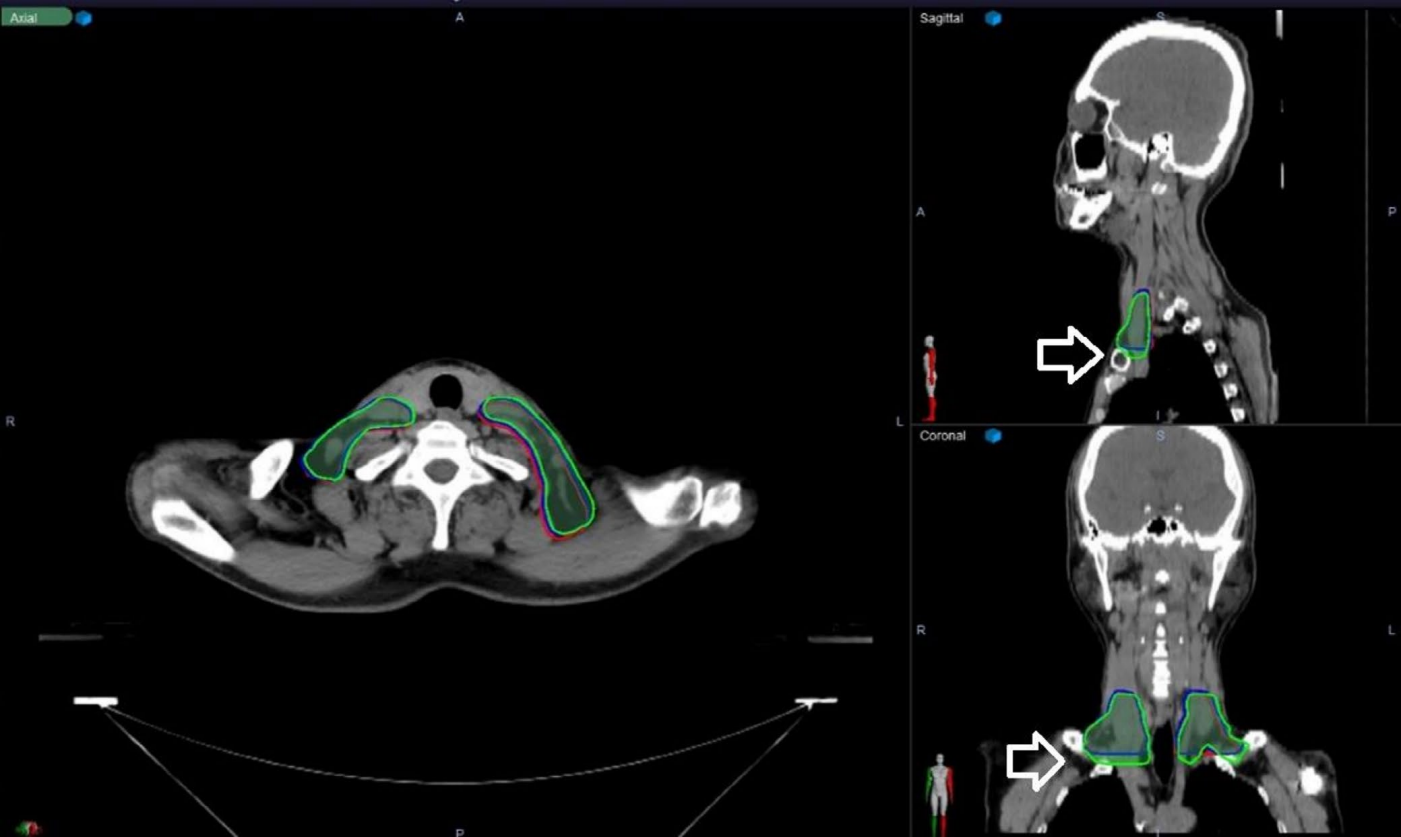
The PTV contours of the prescribed dose of 56 Gy, after 3D (blue) and 6D (red) corrections, are compared with the original plan (green) for a 54‐year‐old male patient with nasopharyngeal cancer at 5 weeks. The white arrows indicate the advantage of 6D correction over 3D correction for PTV‐low contour.

The average volume percentages achieving the 56 Gy prescribed dose before and after 3D and 6D corrections compared to CT‐sim treatment planning are shown in Table 2. The ANOVA analysis demonstrated significant differences in the percentage of target volumes receiving prescribed doses of 56 Gy before and after 3D and 6D corrections (*p* = 0.016). The LSD post hoc multiple comparisons further revealed significant differences before and after 6D corrections, and a significantly higher volume percentage with 6D correction than that with 3D correction at 5 weeks. Figure 3 shows the example of PTV‐low contours after 3D and 6D corrections in a patient with nasopharyngeal cancer. The results also revealed that the PTV‐low contour after 6D correction exhibited higher conformity with the original plan than that after 3D correction at 5 weeks, especially at distal areas.

For the spinal cord, the average percentage changes in Dmax were slightly decreased after 3D and 6D corrections at 1, 3 and 5 weeks, compared to the CT‐sim original plan, as shown in Table 3. However, for the brain stem, the percentage changes in Dmax were generally unchanged after 3D and 6D corrections at 1, 3 and 5 weeks, compared to the CT‐sim original plan. The statistical analysis did not reveal significant differences in Dmax before and after 3D and 6D corrections.

**TABLE 3:** The percentage difference of Dmax before and after 3D and 6D corrections at 1, 3, and 5 weeks in NPC patients.

	Dmax (1 week)	Dmax (3 weeks)	Dmax (5 weeks)
Spinal cord			
Before	8.7% ± 14.5%	8.3% ± 12.4%	8.1% ± 9.7%
3D	5.7% ± 9.2%	5.9% ± 9.5%	6.2% ± 7.7%
6D	5.2% ± 8.2%	4.6% ± 8.9%	0.1% ± 20.2%
Brain stem			
Before	1.4% ± 12.1%	2.9% ± 14.3%	3.6% ± 13.8%
3D	−0.5% ± 8.8%	2.6% ± 11.5%	3.2% ± 8.9%
6D	1.9% ± 5.9%	4.8% ± 10.5%	1.4% ± 7.1%

Moreover, the translations and rotations after 3D and 6D corrections for CBCT images are shown in Table 4. Although no significant differences in translation and rotations were noted between 1, 3 and 5 weeks, the translational errors of the X direction were slightly higher than those of the other two directions using both 3D and 6D corrections. Besides, the rotational errors in the Pitch direction were slightly higher than the other two directions using 6D correction.

**TABLE 4:** The translations (*X*, *Y* and *Z*) and rotations (Raw, Yow and Pitch) after 3D and 6D corrections at 1, 3 and 5 weeks in NPC patients.

	1 week	3 weeks	5 weeks
3D correction			
X (mm)	1.1 ± 1.3	0.4 ± 1.9	1.2 ± 1.9
Y (mm)	0.8 ± 2.1	−0.4 ± 2.8	−0.6 ± 2.7
Z (mm)	−0.4 ± 2.5	−0.6 ± 3.2	−0.7 ± 2.1
6D correction			
X (mm)	1.3 ± 1.7	0.4 ± 2.6	1.2 ± 2.0
Y (mm)	0.7 ± 2.1	−0.3 ± 2.9	−0.7 ± 2.4
Z (mm)	0.2 ± 2.4	0.3 ± 3.1	0.2 ± 1.9
Pitch (degree)	0.8 ± 1.2	1.0 ± 1.3	0.5 ± 1.2
Roll (degree)	−0.4 ± 1.3	−0.2 ± 1.3	−0.5 ± 1.6
Yaw (degree)	−0.1 ± 1.1	−0.0 ± 1.3	0.1 ± 1.2

## 4 Discussion

The present study enrolled 30 patients with nasopharyngeal cancer who received image‐guided radiation therapy, and performed 3D and 6D corrections, respectively, for image registration between CBCT‐ and CT‐sim images. The differences in target volume coverage and the maximum dose to OAR for three prescribed dose levels (70, 63 and 56 Gy) were evaluated using both 3D and 6D corrections. The results demonstrated that the 6D correction achieved approximately 95% dose coverage of the target volume at all three dose levels, while the 3D correction achieved about 94% dose coverage at 70 and 63 Gy. Additionally, the 6D correction provided a significantly higher percentage of dose coverage of the target volume compared to the 3D correction at the 56‐Gy dose level (95.6% ± 5.0% vs. 92.1% ± 7.8%). These findings suggest that the rotational alignment provided by 6D correction is advantageous for covering the elongated target volume of the distal peripheral lymph nodes at the 56‐Gy dose level in nasopharyngeal cancer.

In nasopharyngeal cancer, the nasopharynx is located adjacent to the sphenoid bone, and alignment methods primarily based on the sphenoid bone would preferentially align the nearby nasopharynx [14]. Consequently, errors in the target volumes for 63 Gy and the lower neck region for 56 Gy were likely influenced by the pitch angle of the neck, resulting in greater rotational errors along the *X*‐axis. In our study, the 6D correction, which included both translational and rotational adjustments in the *X*, *Y* and *Z* axes, resulted in significantly higher percentage dose coverage of the target volume compared to the 3D correction at the 56‐Gy dose level. In contrast, the target volume for the prescribed dose of 70 Gy corresponds to the primary tumour site in the nasopharynx, which has a relatively spherical shape. As a result, the percentage coverage of this target volume was less affected by rotational errors, leading to no significant difference between the 3D and 6D corrections at the 70‐Gy dose level.

According to the International Commission on Radiation Units and Measurements (ICRU) Report 50 [15], insufficient or incomplete coverage of the PTV may lead to decreased tumour control rates. The ICRU recommends that at least 95% of the target volume should receive the prescribed dose for effective tumour control. In our study, the average percentage of target volume coverage reaching 95% for the three PTVs (PTV‐high, PTV‐mid, PTV‐low) differed between the 3D and 6D corrections. Of the 30 patients, 14 achieved 95% dose coverage with 3D correction, while 15 achieved this with 6D correction at the 70‐Gy dose level (PTV‐high). For the 63‐Gy dose level (PTV‐mid), 12 patients reached 95% dose coverage with 3D correction, compared to 16 with 6D correction. At the 56‐Gy dose level (PTV‐low), 7 patients achieved 95% dose coverage with 3D correction, while 21 achieved this with 6D correction. These results indicate that 6D correction increased the number of patients achieving 95% coverage of the target tumour across all three PTVs compared to 3D correction.

The overall average percentage of the maximum dose to the brain stem relative to the maximum dose from the CT‐sim treatment planning was higher with 6D correction than with 3D correction (2.3% ± 7.64% vs. 1.4% ± 10.15%), although no significant difference was observed. In the 6D correction, the average rotation angles around the *X*, *Y* and *Z* axes were 0.72° ± 0.23°, −0.44° ± 0.24°, and −0.01° ± 0.07°, respectively. The rotation around the *X*‐axis corresponds to pitch, the rotation around the *Y*‐axis corresponds to roll, and the rotation around the *Z*‐axis corresponds to yaw. Given that the brain stem is located behind the sphenoid bone, adjacent to both the nasopharynx and the slope of the sphenoid bone, it can be inferred that the rotation around the *X*‐axis contributed to the increased dose to the brain stem. In our study, the rotational correction along the *X*‐axis (0.72° ± 0.23°) may have inadvertently placed the brain stem in a high‐dose area, resulting in an increased maximum dose to the brain stem after 6D correction. However, further investigation will be needed to determine whether limiting the angle of *X*‐axis rotation in 6D correction can help reduce the maximum dose to the brain stem without compromising PTV coverage.

One previous study conducted by Rodrigues et al. demonstrated that the 6D couch position correction provided higher inter‐fraction reproducibility than 3D correction, suggesting that 6D correction combined with individual head support reduced inter‐fraction translational and rotational errors [16]. Other previous studies also showed that the 6D correction for CBCT images was helpful to improve the PTV prescription dose coverage for head‐and‐neck, pelvic and thoracic tumours [8, 17]. It was shown that both translational and rotational errors could cause considerably decreased PTV and increased OAR dose in patients with elongated tumours, such as head‐and‐neck and paraspinal tumours [17]. Consistently, our study demonstrated that 6D correction of CBCT images provided significantly higher dose coverage of PTV‐low (56 Gy) than that of 3D correction, further indicating that 6D correction of CBCT images was helpful to improve the dose coverage of PTV‐low. Therefore, it may be possible to consider reducing the PTV‐low margin to minimize side effects on normal neck tissues when using 6D correction for CBCT images [18]. However, since the 6D correction did not significantly reduce the maximum dose to the brainstem, it is suggested to maintain the same OAR margin for the brainstem.

In radiation therapy for nasopharyngeal carcinoma, the priority of protecting critical organs, such as the brainstem, takes precedence over achieving dose coverage of peripheral low‐dose target areas (e.g., PTV‐low, 56 Gy). The present study adopted a more stringent brainstem dose limit indicator of D0.01cc ≤ 54 Gy in the inverse treatment planning design and dose assessment, compared to the standard value of D0.03cc. The results showed that the maximum dose to the brainstem (D0.01cc) for all patients was strictly and safely controlled within the clinical limit of 54 Gy, indicating that improvements in PTV coverage did not come at the expense of exceeding the dose limits for the brainstem. However, when significant anatomical changes occur in patients during the later stages of radiation therapy (such as severe weight loss, atrophy of the parotid glands, or tumour shrinkage), the spatial relationship between the body surface, rigid skeletal structures, and target areas undergoes complex nonlinear deformations, necessitating re‐simulation. In clinical practice, special attention is given to the positioning of head and neck patients in the pitch direction. We have established a clinical protocol stipulating that if the pitch rotation exceeds 1.5 degrees, a physicist must conduct a new dose assessment because the shape of the target area and anatomical structures in the pitch direction are more sensitive to dose distribution changes. However, future investigations will be needed to determine whether 6D correction can provide sufficient dose compensation when patients experience mild to moderate anatomical changes by comparing PTV coverage and maximum dose to OAR between patients with and without re‐simulation.

There are several limitations to this study that warrant discussion. First, the sample size was small, and a larger cohort study may provide more comprehensive results. Second, the registration method used in this study was rigid registration. Although attempts were made to realign the CBCT‐ and CT‐sim images using a deformable approach in the image fusion system, current limitations in radiation therapy hardware prevent patients from undergoing deformable corrections. Third, follow‐up reports were not collected for comparison of late‐stage side effects in patients. Further investigation will be needed to understand the differences in late‐stage side effects between 3D and 6D corrections.

## 5 Conclusion

The present study compared the dose coverage of the target volume and the maximum dose to OAR in patients with nasopharyngeal cancer between 3D and 6D corrections. Compared to 3D correction, the results demonstrated that 6D correction generally improved dose coverage of the target tumour volume at three different dose levels, particularly for PTV‐low, while also slightly reducing the maximum dose to the spinal cord. Therefore, we conclude that 6D correction for CBCT images is beneficial for achieving better dose coverage of the target tumour volume than 3D correction in patients with nasopharyngeal carcinoma during fractionated radiation therapy.

## Funding

This research was supported partly by a grant (NSTC 115‐2314‐B‐037‐046) from the National Science and Technology Council of Taiwan.

## Ethics Statement

This study was approved by the local institutional review board of Kaohsiung Medical University Hospital (KMUHIRB‐E(II)‐20220040).

## Conflicts of Interest

The authors declare no conflicts of interest.

## Data Availability

The data that support the findings of this study are available on request from the corresponding author. The data are not publicly available due to privacy or ethical restrictions.

## References

[1] L. L. Tang , W. Q. Chen , W. Q. Xue , et al., “Global Trends in Incidence and Mortality of Nasopharyngeal Carcinoma,” Cancer Letters 374, no. 1 (2016): 22–30. 10.1016/j.canlet.2016.01.040 26828135

[2] G. Carioli , E. Negri , D. Kawakita , W. Garavello , C. La Vecchia , and M. Malvezzi , “Global Trends in Nasopharyngeal Cancer Mortality Since 1970 and Predictions for 2020: Focus on Low‐Risk Areas,” International Journal of Cancer 140, no. 10 (2017): 2256–2264. 10.1002/ijc.30660 28224615

[3] Y. T. Zhang , H. Rumgay , M. M. Li , S. M. Cao , and W. Q. Chen , “Nasopharyngeal Cancer Incidence and Mortality in 185 Countries in 2020 and the Projected Burden in 2040: Population‐Based Global Epidemiological Profiling,” JMIR Public Health and Surveillance 9 (2023): e49968. 10.2196/49968 PMC10551785 37728964

[4] F. Bray , M. Laversanne , H. Y. A. Sung , et al., “Global Cancer Statistics 2022: GLOBOCAN Estimates of Incidence and Mortality Worldwide for 36 Cancers in 185 Countries,” CA: A Cancer Journal for Clinicians 74, no. 3 (2024): 229–263. 10.3322/caac.21834 38572751

[5] P. Bossi , A. T. Chan , L. Licitra , et al., “Nasopharyngeal Carcinoma: ESMO‐EURACAN Clinical Practice Guidelines for Diagnosis, Treatment and Follow‐Up,” Annals of Oncology 32, no. 4 (2021): 452–465. 10.1016/j.annonc.2020.12.007 33358989

[6] R. Jiromaru , T. Nakagawa , and R. Yasumatsu , “Advanced Nasopharyngeal Carcinoma: Current and Emerging Treatment Options,” Cancer Management and Research 14 (2022): 2681–2689. 10.2147/CMAR.S341472 PMC9480178 36117730

[7] J. Wang , S. Bai , N. Y. Chen , et al., “The Clinical Feasibility and Effect of Online Cone Beam Computer Tomography‐Guided Intensity‐Modulated Radiotherapy for Nasopharyngeal Cancer,” Radiotherapy and Oncology 90, no. 2 (2009): 221–227. 10.1016/j.radonc.2008.08.017 18930327

[8] W. Fu , Y. Yang , N. J. Yue , D. E. Heron , and S. M. Huq , “Study of Rotational Setup Errors and Their Dosimetric Impacts on Head and Neck IMRT Treatments Using Kilovoltage Cone‐Beam Computed Tomography (kV CBCT),” International Journal of Radiation Oncology, Biology, Physics 69, no. 3 (2007): S188.

[9] T. Singh , D. Singh , S. C. Murphy , A. Bin Sumaida , and N. M. Shanbhag , “Initial Experience With 6D Skull Tracking and Intrafractional Motion Monitoring in The United Arab Emirates' First CyberKnife(R) Radiosurgery Center,” Cureus 16, no. 1 (2024): e52143. 10.7759/cureus.52143 PMC10784719 38222986

[10] S. Lei , N. Piel , E. K. Oermann , et al., “Six‐Dimensional Correction of Intra‐Fractional Prostate Motion With CyberKnife Stereotactic Body Radiation Therapy,” Frontiers in Oncology 1 (2011): 48. 10.3389/fonc.2011.00048 PMC3356099 22655248

[11] X. Sun , Z. Dai , M. Xu , X. Guo , H. Su , and Y. Li , “Quantifying 6D Tumor Motion and Calculating PTV Margins During Liver Stereotactic Radiotherapy With Fiducial Tracking,” Frontiers in Oncology 12 (2022): 1021119. 10.3389/fonc.2022.1021119 PMC9709257 36465406

[12] H. S. Choi , K. M. Kang , I. B. Ha , et al., “Comprehensive Analysis of Set‐Up Gain of 6‐Dimensional Cone‐Beam CT Correction Method in Radiotherapy for Head and Neck and Brain Tumors,” Computational and Mathematical Methods in Medicine 2022 (2022): 2964023. 10.1155/2022/2964023 PMC9613383 36311255

[13] J. L. Ma , Z. Chang , Z. H. Wang , Q. J. Wu , J. P. Kirkpatrick , and F. F. Yin , “ExacTrac X‐Ray G Degree‐Of‐Freedom Image‐Guidance for Intracranial Non‐Invasive Stereotactic Radiotherapy: Comparison With Kilo‐Voltage Cone‐Beam CT,” Radiotherapy and Oncology 93, no. 3 (2009): 602–608. 10.1016/j.radonc.2009.09.009 19846229

[14] T. Cheo , Y. Loh , D. Chen , K. M. Lee , and I. Tham , “Measuring Radiotherapy Setup Errors at Multiple Neck Levels in Nasopharyngeal Cancer (NPC): A Case for Differential PTV Expansion,” Radiotherapy and Oncology 117, no. 3 (2015): 419–424. 10.1016/j.radonc.2015.09.032 26603773

[15] International Commission on Radiation Units and Measurements (ICRU) , “Report 50,” https://www.icru.org/report/prescribing‐recording‐and‐reporting‐photon‐beam‐therapy‐report‐50/. 5420576

[16] M. F. Rodrigues , S. Veen , J. van Egmond , et al., “The Influence of a Six Degrees of Freedom Couch and an Individual Head Support in Patient Positioning in Radiotherapy of Head and Neck Cancer,” Physics and Imaging in Radiation Oncology 11 (2019): 30–33. 10.1016/j.phro.2019.07.001 PMC7807734 33458274

[17] M. Guckenberger , J. Meyer , D. Vordermark , K. Baier , J. Wilbert , and M. Flentje , “Magnitude and Clinical Relevance of Translational and Rotational Patient Setup Errors: A Cone‐Beam CT Study,” International Journal of Radiation Oncology, Biology, Physics 65, no. 3 (2006): 934–942. 10.1016/j.ijrobp.2006.02.019 16751076

[18] D. S. Pruthi , P. Nagpal , and M. Pandey , “Effectiveness of 6D Couch With Daily Cone Beam Computed Tomography in Reducing PTV Margins for Glioblastoma Multiforme,” Journal of Neurosciences in Rural Practice 14, no. 1 (2023): 78–83. 10.25259/JNRP_2_2022 PMC9943941 36891114

